# Crosstalk between cytotoxic CD8+ T cells and stressed cardiomyocytes triggers development of interstitial cardiac fibrosis in hypertensive mouse hearts

**DOI:** 10.3389/fimmu.2022.1040233

**Published:** 2022-11-22

**Authors:** Kurt Brassington, Peter Kanellakis, Anh Cao, Ban-Hock Toh, Karlheinz Peter, Alex Bobik, Tin Kyaw

**Affiliations:** ^1^ Inflammation and Cardiovascular Disease Laboratory, Baker Heart and Diabetes Institute, Melbourne, VIC, Australia; ^2^ Centre for Inflammatory Diseases, Department of Medicine, Monash Medical Centre, Clayton, VIC, Australia; ^3^ Baker Department of Cardiometabolic Health, University of Melbourne, Parkville, VIC, Australia; ^4^ Department of Immunology, Monash University, Melbourne, VIC, Australia

**Keywords:** cardiac fibrosis, CD8+ T cells, perforin, NKG2D, cardiomyocytes, RAE-1, STING, TGF-β1

## Abstract

**Aims:**

Cardiac fibrosis is central to heart failure (HF), especially HF with preserved ejection fraction (HFpEF), often caused by hypertension. Despite fibrosis causing diastolic dysfunction and impaired electrical conduction, responsible for arrhythmia-induced sudden cardiac death, the mechanisms are poorly defined and effective therapies are lacking. Here we show that crosstalk between cardiac cytotoxic memory CD8+ T cells and overly stressed cardiomyocytes is essential for development of non-ischemic hypertensive cardiac fibrosis.

**Methods and results:**

CD8 T cell depletion in hypertensive mice, strongly attenuated CF, reduced cardiac apoptosis and improved ventricular relaxation. Interaction between cytotoxic memory CD8+ T cells and overly stressed cardiomyocytes is highly dependent on the CD8+ T cells expressing the innate stress-sensing receptor NKG2D and stressed cardiomyocytes expressing the NKG2D activating ligand RAE-1. The interaction between NKG2D and RAE-1 results in CD8+ T cell activation, release of perforin, cardiomyocyte apoptosis, increased numbers of TGF-β1 expressing macrophages and fibrosis. Deleting NKG2D or perforin from CD8+ T cells greatly attenuates these effects. Activation of the cytoplasmic DNA-STING-TBK1-IRF3 signaling pathway in overly stressed cardiomyocytes is responsible for elevating RAE-1 and MCP-1, a macrophage attracting chemokine. Inhibiting STING activation greatly attenuates cardiomyocyte RAE-1 expression, the cardiomyocyte apoptosis, TGF-β1 and fibrosis.

**Conclusion:**

Our data highlight a novel pathway by which CD8 T cells contribute to an early triggering mechanism in CF development; preventing CD8+ T cell activation by inhibiting the cardiomyocyte RAE-1-CD8+ T cell-NKG2D axis holds promise for novel therapeutic strategies to limit hypertensive cardiac fibrosis.

## Graphical Abstract

Cardiac fibrosis developed from fibrotic remodelling due to hypertension leads to heart failure. Immune cells are abundantly accumulated in fibrotic regions, but exact mechanisms how they contributed to cardiac fibrosis are not clear. Here we demonstrated that CD8 T cells are major contributor to cardiac fibrosis in hypertensive hearts. Stressed cardiomyocytes express STING-dependent RAE-1 that activates NKG2D + CD8 T cells to induce apoptosis of stressed cardiomyocytes. Preventing STING signalling in stressed cardiomyocytes attenuates cardiac fibrosis. Pharmacologically inhibiting cardiomyocayte-RAE-1 and CD8+ T cell-NKG2D axis may be a potential therapeutic strategy to prevent cardiac fibrosis in HFpEF patients.

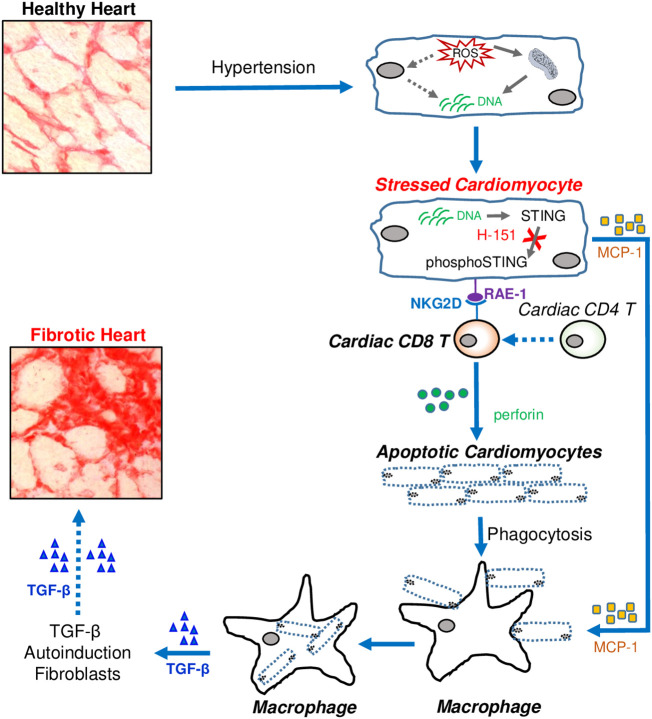

## Introduction

The accumulation of fibrotic tissue seen as a diffuse increase in collagen fibers accounts for most of the fibrotic remodeling of the heart during hypertension (1, 2), the most common risk factor for heart failure with preserved ejection fraction (HFpEF) (3), and a leading cause of death (4, 5). Fibrosis largely accounts for the poor cardiac function in HFpEF, greatly impairing ventricular relaxation; it also increases the frequency of life-threatening arrhythmias (6, 7). Transforming growth factor-beta1 (TGF-β1) is the most important pro-fibrotic cytokine for development of cardiac fibrosis (8) and exerts its effects by stimulating cardiac fibroblasts to produce collagen (9, 10). However, despite its importance, neither it nor its receptors are suitable therapeutic targets for fibrosis, as inhibition leads to development of severe autoimmune-like symptoms, a consequence of its many pleiotropic effects (11).

Immune cells are also potentially important therapeutic targets with mast cells (12), macrophages (13), CD4+ and CD8+ T cells (14) strongly associated with cardiac fibrosis. Cardiac fibrosis is greatly reduced in TCR-α-/- mice, indicating a critically important role for T cells (15), but the role of CD8+ T cells in hypertensive cardiac fibrosis remains unclear. Using mice deficient in CD8α chain Laroumanie et al. concluded that CD8+ T cells were not important for development of cardiac fibrosis (16). In contrast studies by Ma et al, also using mice deficient in CD8α chain, concluded that CD8+ T cells are critically important for cardiac fibrosis (17). Furthermore, using MHC class I-restricted OVA-specific TCR transgenic mice, these investigators concluded that CD8+ T cells exert pro-fibrotic effects *via* yet-to-be-defined innate TCR-independent mechanisms (17, 18).

In an attempt to resolve this controversy, we chose to examine the effects of targeting CD8β chain rather than the α chain; most CD8+ T cells express a heteromeric CD8 consisting of α- and β-chains (19). We examined the effects of deleting CD8β chain on cardiac fibrosis in three different hypertensive models: mice with renal hypertension, mice with steroid dependent hypertension and mice with systolic hypertension initiated by aortic constriction. We also define the mechanisms by which CD8+ T cells contribute to hypertensive fibrosis. We initially confirmed the earlier finding of Ma et al (17) that CD8+ T cells are critical for development of hypertensive cardiac fibrosis and also demonstrated that this profibrotic effect of CD8+ T cells is independent of mechanisms responsible for elevating blood pressure.

We further extended the earlier finding of Ma et al. on innate TCR and development of hypertensive cardiac fibrosis (17), demonstrating that crosstalk between CD8+ T cells and overly stressed cardiomyocytes is critical for development of fibrosis. CD8+ T cells that accumulate in fibrotic heart express the cytotoxin perforin and the innate receptor NKG2D that recognizes stress-induced surface ligands and is sufficient to initiate a killing response (20). Stressed cardiomyocytes express the NKG2D receptor activating ligand RAE-1 as well as monocyte chemoattractant protein-1 (MCP-1) initiated *via* the cGAS-STING cytosolic DNA sensing pathway. Interaction of NKG2D with RAE-1 activates the cytotoxic functions of CD8+ T cells resulting in killing of overly stressed cardiomyocytes *via* apoptosis, whilst MCP-1 attracts macrophages. Clearance of apoptotic cells by macrophages is known to increase their expression of TGF-β1 (20) that may initiate the fibrotic process by stimulating collagen production by fibroblasts (21); we found increased numbers of macrophages expressing TGF-β within fibrotic regions. TGF-β1 by interacting with fibroblasts further amplifies this effect as it positively regulates its own expression (22, 23).

Our results uncover an unexpected unique mechanism for development of hypertensive cardiac fibrosis involving specific interactions between innate cytotoxic CD8+ T cells and stressed cardiomyocytes that may lead to development of new therapeutic strategies to prevent hypertensive cardiac fibrosis associated with HFpEF.

## Materials and methods

### Animals, ethics and experiments

Experiments were approved by the Animal Ethics Committee at the Alfred Research Alliance, Melbourne, Australia. All mice were on a C57BL/6 genetic background. NKG2D knockout (NKG2D^-/-^) mice were from Jackson Laboratory, Bar Harbor, Maine, USA; perforin knockout (pfp^-/-^) mice were from Mark Smyth, Peter MacCallum Cancer Centre, Melbourne, Australia, CD8β knockout mice were from David Davis, Australian National University, Canberra, Australia with approval from Dan Littman, New York School of Medicine, USA and wild-type C57BL/6 mice were from WEHI, Melbourne, Australia. Mixed chimeric mice whose CD8+ T cells were deficient in perforin or NKG2D were generated using our previously published procedure (24). Briefly, 6-week-old male wildtype C57BL/6 mice were irradiated with 1100 Gy. To generate mixed chimeric mice with perforin-deficiency limited to CD8+ T cells, irradiated mice were transplanted with 5 million mixed bone marrow cells from CD8β-knockout (80%) and perforin-deficient mice (20%); control mice received bone marrow from CD8β-knockout mice (80%) and wild type mice (20%). An identical strategy was used to generate chimeric mice with NKG2D-deficiency limited to CD8+ T cells. Four weeks after transplantation mice were subjected to TAC surgery. Bone marrow reconstitution and specific perforin/NKG2D deficiencies were confirmed by FACS/immunofluorescence.

Ketamine 80mg/Kg, Xylazine 20mg/Kg and Atropine 0.06mg/Kg (via intraperitoneal injection) were used to induce a short anesthesia for surgical procedures. Hypertension/pressure overload was induced by trans-aortic constriction (TAC) of the thoracic aorta reducing aortic lumen to 0.44mm as previously described by us (12). In Sham mice the aortic arch was not narrowed. Two-kidney one-clip (2K1C) hypertension was also induced in anaesthetized mice. The left renal artery exposed and a silver clip (120 µm gap) placed around the artery (25). Sham operated mice did not undergo clipping. For unilateral nephrectomy-DOCA-salt (1K-DOCA-salt) hypertension the left kidney was removed and a deoxycorticosterone acetate (DOCA) pellet (2.4 mg/day, 21 days; Innovative Research of America, USA) implanted subcutaneously (26). Sham mice did not receive DOCA. These mice were given 0.9% saline as drinking water.

Blood pressure and left ventricular diastolic function were measured by micromanometry under anaesthesia [ketamine, xylazine and atropine (80/20/0.06 mg/kg i.p.)] whilst mice were ventilated 28 days post-surgeries using a 1.4F Millar microtipped transducer catheter (Millar Instruments, Houston, TX, USA). The Miller catheter was then inserted into the aorta and the aortic blood pressure recorded using a computer and AD Instrument software (AD Instruments, Australia). The catheter was then advanced into the left ventricle to assess left ventricular diastolic function; –dp/dt (min), relaxation time constant (*Tau*), and left ventricular end-diastolic pressure (LVEDP) as previously described (27).

For CD8+ T cell depletion, the rat anti-mouse CD8β (lyt-3) monoclonal antibody YTS 156.7 prepared from a hybridoma provided by Steve Cobbold, Oxford University, UK. Mice were administered 0.5 mg antibody/mouse/week i.p, for 4 weeks; IgG2b isotype was administered to control mice. CD4+ T cells were depleted using an anti-CD4 monoclonal antibody (GK1.5) 0.3mg/week i.p. and controls were given non-immune IgG2b. For STING inhibition mice were administered H-151 (0.21mg/day i.p.) commencing one day after surgery for 4 weeks; H-151 covalently blocks STING palmitoylation preventing TBK1 activation and STING phosphorylation (28, 29).

At the end of experiment, mice were euthanized using the gradual-fill method of carbon dioxide overdose followed by cervical dislocation. However, mice anesthetised for hemodynamic measurements were euthanized *via* cervical dislocation.

### Flow cytometry

Flow cytometry was performed as previously described (24), using APC-conjugated rat anti-mouse CD4 mAb (clone: RM4-5; BD Biosciences), PerCP conjugated anti-mouse CD8α mAb (clone: 53-6.7; BD Biosciences), APC-Cy7conjugated Armenian hamster anti-mouse TCR-β mAb (clone: H57-597; Biolegend) and FITC conjugated rat anti-mouse CD19 antibody (BD Pharmingen). For perforin staining PE conjugated rat anti-mouse perforin (clone: eBioOMAK-D; eBiosciences) was used. For intracellular perforin, spleen cells were stimulated for 12 hours with phorbol 12-myristate 13-acetate, ionomycin, brefeldin A and monensin (eBioscience, San Diego, CA). A BD LSRFortessa was used to acquire flow cytometry data, analysed using BD FACSDiva.

### Cardiac tissue processing

The left ventricle (LV) was dissected into three equal transverse LV slices and frozen in OTC at -70°C; 6 µm-thick sections were cut for histology/immunofluorescence stainings and 30 µm-thick sections for ROS assessment.

### Cardiac fibrosis

For collagen deposition sections were stained with Picrosirius Red F3BA, (0.1% solution in saturated aqueous picric acid) and analysed using a computer –interfaced colour Imaging system (Optimus Bioscan 2. Thomas Optical Measurement System, Inc). Ten randomly selected fields from each section were analysed and expressed as percent of the stained area (12, 14).

### Immunohistochemical staining

Six µm sections were allowed to thaw then fixed in cold (-20°C) acetone for 20 min. Sections were sequentially incubated in 3% hydrogen peroxide in PBS (20 min), 10% normal serum/PBS (30 min) and biotin/avidin-blocking reagents (15 min each reagent) (Vector Laboratories). These sections were then incubated (1hr) with primary antibodies in serum: rat anti-mouse CD4 (BD: cat#550280), rat anti-mouse CD8 (BD: cat#550281), rat anti-mouse CD68 (BioRad: cat#MCA1957), rabbit anti-mouse TGFβ1 (Novus Biological: cat#NB100-91995), or corresponding non-immune IgG’s. Then, sections were washed and incubated with the appropriate secondary antibody (biotinylated mouse anti-rat: BD Pharmingen cat#550325 or biotinylated anti-rabbit; Vector Labs: cat#BA-1000)] for 40 min, followed by incubation with streptavidin horseradish peroxidase complex (30 min) (Vector Laboratories) and counterstaining with haematoxylin. Antigens were visualised using 3, 3-diamonobenzidine (Sigma). Cell expressing antigens were quantitated by cell counting in ten randomly selected fields per section.

### Immunofluorescence

Sections were fixed in acetone, washed and permeabilized in 0.1% Triton-X-100/PBS. After washings, sections were incubated in 10% normal serum/PBS before being incubated with primary antibodies in serum: rabbit anti-mouse CD8 (Sino Biological: cat#50389-R20), mouse anti-mouse NK1.1 (Biolegend: cat#108701), Armenian Hamster- anti-mouse TCR γ/δ (Biolegend: cat#108101), rat anti-mouse Perforin (LS Biosciences: cat#LS-C18632), biotin rat anti-mouse NKG2D (R&D Systems; cat#BAM1547), rabbit anti-mouse TGFβ1 (Novus Biological: cat#NB100-91995), rat anti-mouse CD68 (BioRad: cat#MCA1957), rabbit anti-mouse troponin (Abcam: cat#ab125266), goat anti-mouse RAE-1 (R&D Systems: cat#AF1136), rabbit anti-mouse α-Actinin (Acris Antibodies: cat#BP210), rabbit anti-mouse phosphor-STING (Invitrogen: cat# PA5-105674), goat anti-mouse CX3CR1 (R & D Systems: cat#AF5825), mouse anti-mouse phospho p38MAPK (Biolegend: cat#690201), rabbit anti-mouse phospho-IRF3 (Cell Signaling: cat#29047), rabbit anti-mouse phospho-TBK1 (Imgenex: cat# IMX-5194), mouse anti-mouse NF-kB (Santa Cruz: cat# sc-109), rabbit anti-mouse MCP-1 (Abcam: cat #ab7202), rabbit anti-mouse α-Actinin Biotin (Bioss antibodies: cat# BS-10367R-Biotin) or corresponding isotype matched control IgG antibodies. Then sections were then washed and incubated with secondary antibodies/conjugates (goat anti-rat Alexa 488, Invitrogen: cat#A11006; goat anti-rabbit Alexa 488, Invitrogen: cat#A11034; goat anti-rabbit TRITC, Novex: cat# A24542; goat anti-rat TRITC, Novex: cat#A18870; donkey anti-goat TRITC, Invitrogen: cat#A16004 or Streptavidin Alexa 488, Invitrogen: cat#S11223; goat anti-mouse Alexa 488, Invitrogen: cat# A11029; goat anti-rabbit Alexa 568, Invitrogen: cat#A11036 or streptavidin TRITC, Serotec: cat# STAT3B) for 30 min, washed in PBS and counterstained with 4’,6-diamidino-2-phenylindole dihydrochloride (DAPI). For dual fluorescence stainings sections were first incubated with the first primary antibody/secondary antibody and after multiple washings were incubated with the second primary antibody and its corresponding secondary antibody. Antigens were assessed using an Olympus BX61 microscope with a FV11 CCD camera using cellSens imaging software (Olympus).

### TUNEL staining

Apoptotic cells were identified using a deoxynucleotidyl transferase dUTP nick end labelling (TUNEL) system [*In Situ* Cell Death Detection Kit-POD (Roche: cat#11-684-817-910)]. Briefly, sections were incubated in 4% diethyl pyrocarbonate (DEPC)/100% ethanol at 4C, washed and fixed in acetone. Sections were permeabilized in 0.1% Triton-X-100 and incubated in 15% FCS/2% BSA/0.1M Tris-HCl buffer and after washing, they were incubated with a TUNEL reaction mixture at 37C (according to the manufacturer’s instructions), washed and counterstained with DAPI. TUNEL positive cells were visualized using Olympus BX61 microscope. For double immunofluorescence to identify apoptotic cardiomyocytes frozen sections were stained by TUNEL followed by rabbit anti-mouse α-Actinin (Acris Antibodies, Cat#BP210). Then goat anti-rabbit Alexa 568 (Invitrogen, cat#A11036) was used to detect Actinin. Nuclei were counterstained with DAPI and apoptotic cardiomyocytes visualised using a Nikon A1r confocal microscope. Ten randomly selected fields per section were analysed.

### Cardiomyocyte cytosolic DNA

Cytosolic DNA and cardiomyocytes were stained with Pico488 (picogreen), a highly sensitive fluorescent dye that can detect double stranded cytoplasmic DNA (30, 31) and anti-α-Actinin antibody. Six µm formalin-fixed permeabilized sections of left ventricle were incubated with Pico488 DNA quantification solution (Lumiprobe, Cat#12010) followed by polyclonal rabbit anti-mouse α-Actinin antibody (see above). Then goat anti-rabbit Alexa 568 secondary antibody (see above) was used to detect Actinin. After counterstaining with DAPI the sections were visualised using a Nikon A1r confocal microscope.

### Reactive oxygen species

Reactive oxygen species (ROS) were measured using dihydroethidium (DHE). Sections were incubated with DHE (10 μM, Krebs bicarbonate buffer), protected from light at 37°C for 30 min. Images were obtained using a fluorescence microscope measuring fluorescence *via* a 585 nm long-pass filter (12).

### Statistical analysis

Randomized and coded animals and samples were processed and analyzed by blinded investigators. All data was analyzed statistically using Prism 8.0 (GraphPad Software, La Jolla, CA, USA). All results are expressed as man ± SEM. *n* represents mouse numbers in each experiment, as detailed in figure legends. Comparison between two groups were determined by two-tailed Student’s *t-*test, and three or more groups were compared by one-way ANOVA followed by Tukey-Kramer *post hoc* analysis. *P <* 0.05 was considered statistically significant.

## Results

### CD8+ T cells are critical for hypertensive cardiac interstitial fibrosis irrespective of hypertension etiology

**Figure 1:**
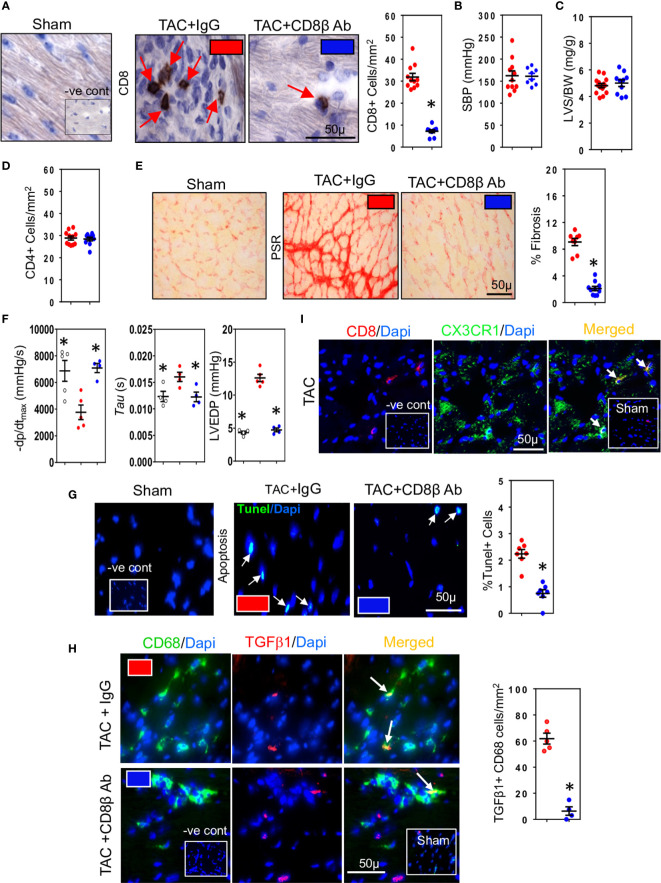
CD8+ T cells promote hypertension-induced cardiac fibrosis. **(A)** Representative photomicrographs of CD8+ T cells within the left ventricle (LV) of hypertensive TAC mice and their depletion using anti-CD8β antibodies. **(B)** Systolic aortic blood pressures (SBP) recorded in aortas by micromanometry and **(C)** LV hypertrophy measured as wall plus septum/body weight (LVS/BW) ratio in TAC mice with/without CD8αβ+ T cells. **(D)** CD4+ T cells in LV are unaffected by CD8αβ+ T cell depletion. **(E)** Photomicrographs showing the reduction in LV fibrosis in TAC mice associated with CD8αβ+ T cell depletion. **(F)** Effects of CD8αβ+ T cell depletion on LV diastolic function (-dp/dt, Tau and LVEDP) measured by micromanometry. **(G)** Photomicrographs showing reductions in TUNEL+ (apoptotic) cells in LV following CD8αβ+ T cell depletion. **(H)** Photomicrographs depicting effects of CD8αβ+ T cell depletion on CD68+ macrophages expressing TGF-β1 in LV of TAC mice. **(I)** Photomicrographs showing CD8+ T cells accumulated in TAC left ventricle expressing CX3CR1. Results are means ± SEM with small circles representing data from individual mice. n=6-12/group. PSR; picrosirius red, -ve control; no primary antibody control, sham; sham-operated, scale bar represents 50µm. *P < 0.05 using two-tailed Student’s t-test or one-way ANOVA comparing to TAC-IgG.

We used three different hypertensive mouse models to assess the importance of CD8+ T cells for development of cardiac fibrosis: 1) mice whose systolic blood pressure is elevated by trans-aortic constriction (TAC) inducing left ventricular pressure overload 2) mice with high-renin salt-independent 2K1C renovascular hypertension and 3) mice with salt-sensitive low-renin deoxycorticosterone (DOCA)-salt hypertension. We used highly specific anti-CD8β antibodies to deplete CD8αβ+ T (CD8+ T) cells (32). Their depletion during the 4-week study period reduced ventricular CD8+ T cells in all three models (**Figures 1A**, **S1A, G**). Compared to normotensive sham-operated mice (Systolic Blood Pressure (SBP) = 103 ± 5 mmHg; n=10), mice subjected to TAC, 2K1C or DOCA procedure were hypertensive (**Figures 1B**, **S1B, H**). Neither hypertension (**Figures 1B**, **S1B, H**), left ventricular hypertrophy (**Figures 1C**, **S1C, I**) nor cardiac CD4+ T cells were affected (**Figure 1D**) but left ventricular fibrosis was significantly reduced (**Figures 1E**, **S1D, S1J**). As fibrosis is associated with poor cardiac function due to impaired ventricular relaxation, we also assessed using micromanometry how CD8+ T cell depletion affected left ventricular diastolic function and relaxation (33). CD8+ T cell depletion greatly improved left ventricular relaxation (-dp/dt) and reduced isovolumic relaxation constant (tau) and left ventricular end diastolic pressure (LVEDP) (**Figures 1F**, **S1E, K**). Since CD8+ T cells can be cytotoxic, we assessed effects on apoptotic cells. CD8+ T Cell depletion reduced cardiac apoptotic cell numbers by 70-79% (**Figures 1G**, **S1F, L**). As macrophages are also important for fibrosis (34), and known to remove apoptotic cells by phagocytosis (35), thus increasing their biosynthesis of TGF-β1 (14, 21), we also assessed macrophage TGF-β1 expression in the TAC mice. TGF-β1-positive macrophages were most apparent in fibrotic regions and greatly reduced following CD8+ T cell depletion (**Figure 1H**). These findings indicate that TGF-β1 produced by macrophages is an early signaling event; this TGF-β1, by activating fibroblasts to produce collagen can further elevate TGF-β1 levels by positively regulating its own expression in fibroblasts (22, 23), greatly increasing the pro-fibrotic signal. To determine whether the CD8+ T cells exhibited a memory phenotype, we assessed their expression of CX_3_CR1. The majority of CD8+ T cells in fibrotic areas of TAC hearts expressed CX_3_CR1 (**Figure 1I**), consistent with a memory CD8+ T cell subset that possesses less proliferative ability, but higher cytotoxic effector function (36). Given the commonality between cardiac fibrosis, CD8+ T cells and cardiac apoptosis in the different hypertensive mice, we focused our subsequent mechanistic studies only on TAC mice.

### CD4+ T cells are required for optimum cardiac CD8+ T cell profibrotic activity

**Figure 2:**
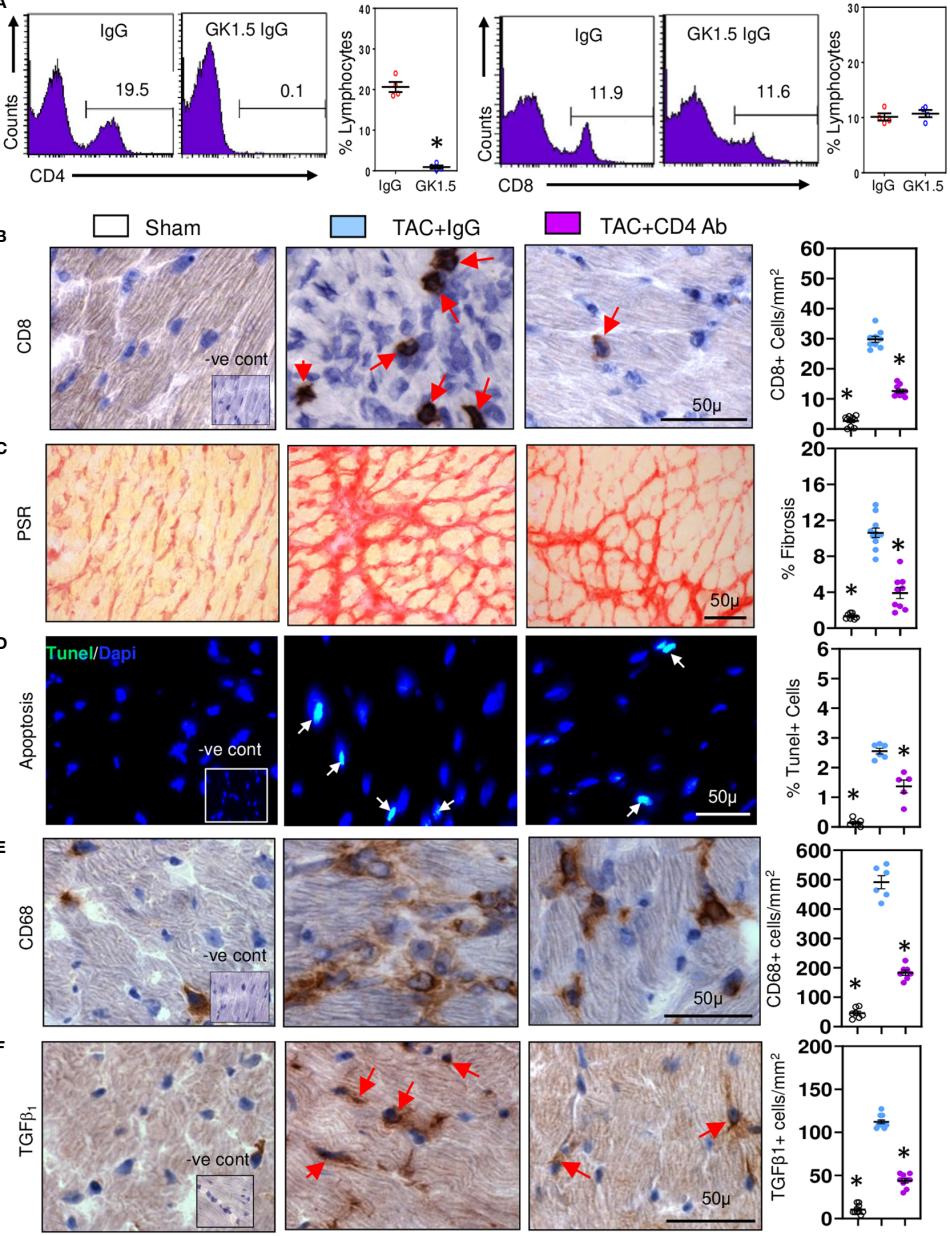
CD8+ T cells require help from CD4+ T cell in promoting cardiac fibrosis. **(A)** Representative FACS plots showing treatment of TAC mice with anti-CD4 antibodies depletes spleen CD4+ cells without affecting CD8+ cells controls. **(B)** Depletion of CD4+ T cells significantly reduces ventricular CD8+ T numbers compared to controls during fibrosis in TAC mice. **(C)** LV fibrosis staining is greatly reduced following CD4+ T cell depletion in TAC mice. **(D)** TUNEL+ cells are reduced by CD4+ T cell depletion in TAC mice. **(E)** CD68+ macrophage numbers are reduced with CD4+ T cell depletion. **(F)** Numbers of TGF-β1 expressing cells in LV are reduced with anti-CD4 antibody treatment. Results are means ± SEM with small circles representing data from individual mice. n=4-9/group. PSR; picrosirius red, -ve control; no primary antibody control, scale bar represents 50µm. *P< 0.05 using two-tailed Student’s t-test one-way ANOVA comparing to TAC-IgG.

As CD4+ T cells contribute to development of cardiac fibrosis (16), we examined whether CD4+ T cells provide help to CD8+ T cells. CD8+ T cells initiating low inflammatory responses are known to be highly dependent on CD4+ T cell help (37). We depleted CD4+ T cells in TAC mice using anti-CD4 cell depleting antibodies and 28 days later examined effects on CD8+ T cells and pro-fibrotic responses. Treatment with depleting antibodies reduced spleen CD4+ cell numbers by approximately 95% without effecting CD8+ cells (**Figure 2A**). In contrast cardiac CD8+ T cells were reduced by 55% (**Figure 2B**) and fibrosis by 60% (**Figure 2C**); apoptotic cell numbers were also reduced, by 45% (**Figure 2D**). Cardiac macrophage numbers were reduced (**Figure 2E**), as were cells expressing TGF-β1 (**Figure 2F**). These findings indicate that CD4+ T cells enhance the profibrotic actions of CD8+ T cells, mostly likely by increasing their survival (38).

### Profibrotic effects of CD8+ T cells are dependent on the cytotoxin perforin and innate NKG2D receptor

**Figure 3:**
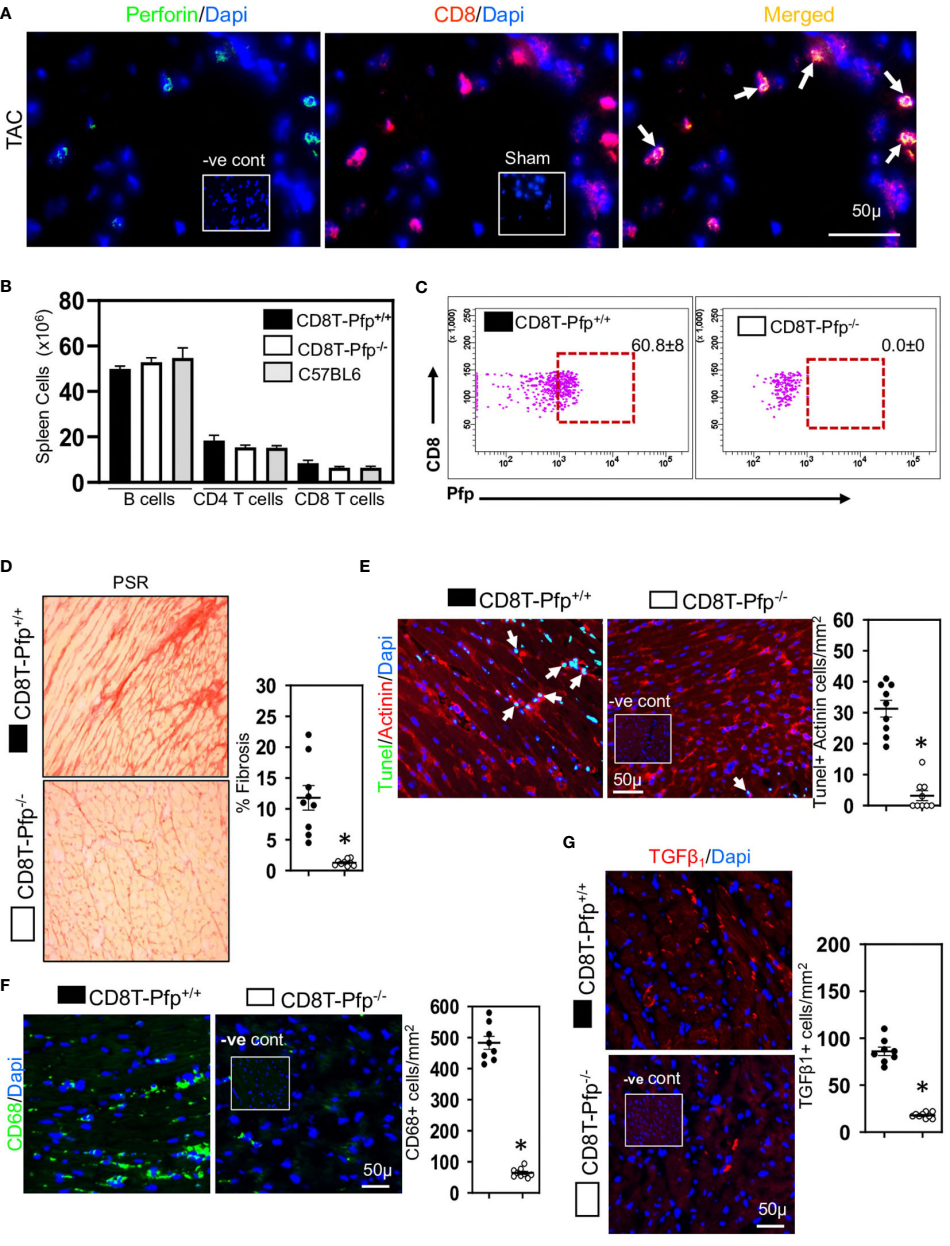
Cardiac CD8+ T cells require perforin to promote fibrosis. **(A)** Representative photomicrographs indicating perforin expression by CD8+ T cells in fibrotic ventricle of TAC mice. **(B)** Bar graphs indicating that 4-weeks after bone marrow transplantation major lymphocyte populations have recovered to levels seen in non-irradiated C57BL/6 mice. **(C)** Representative FACS plots indicating that spleen CD8+ T cells in CD8T-Pfp^-/-^ mice are deficient in perforin 4 weeks after BMT compared to CD8T-Pfpf^+/+^ mice. **(D)** Photomicrographs (PSR) together with mean data indicating that perforin deletion in CD8+ T cells attenuates fibrosis in LV of TAC mice compared to controls. **(E)** Photomicrographs indicating that TUNEL+Actinin+ cardiomyocytes are greatly reduced in mice with perforin deficient CD8+ T cells. **(F)** LV CD68+ macrophages and **(G)** TGF-β1 expressing cells are greatly reduced with perforin deletion from CD8+ T cells. Results are means ± SEM with small circles representing data from individual mice. n=5-9/group. PSR; picrosirius, -ve control; no primary antibody control, sham; sham-operated, scale bar represents 50µm. *P< 0.05 using two-tailed Student’s t-test comparing to CD8T-Pfp^+/+^.

**Figure 4:**
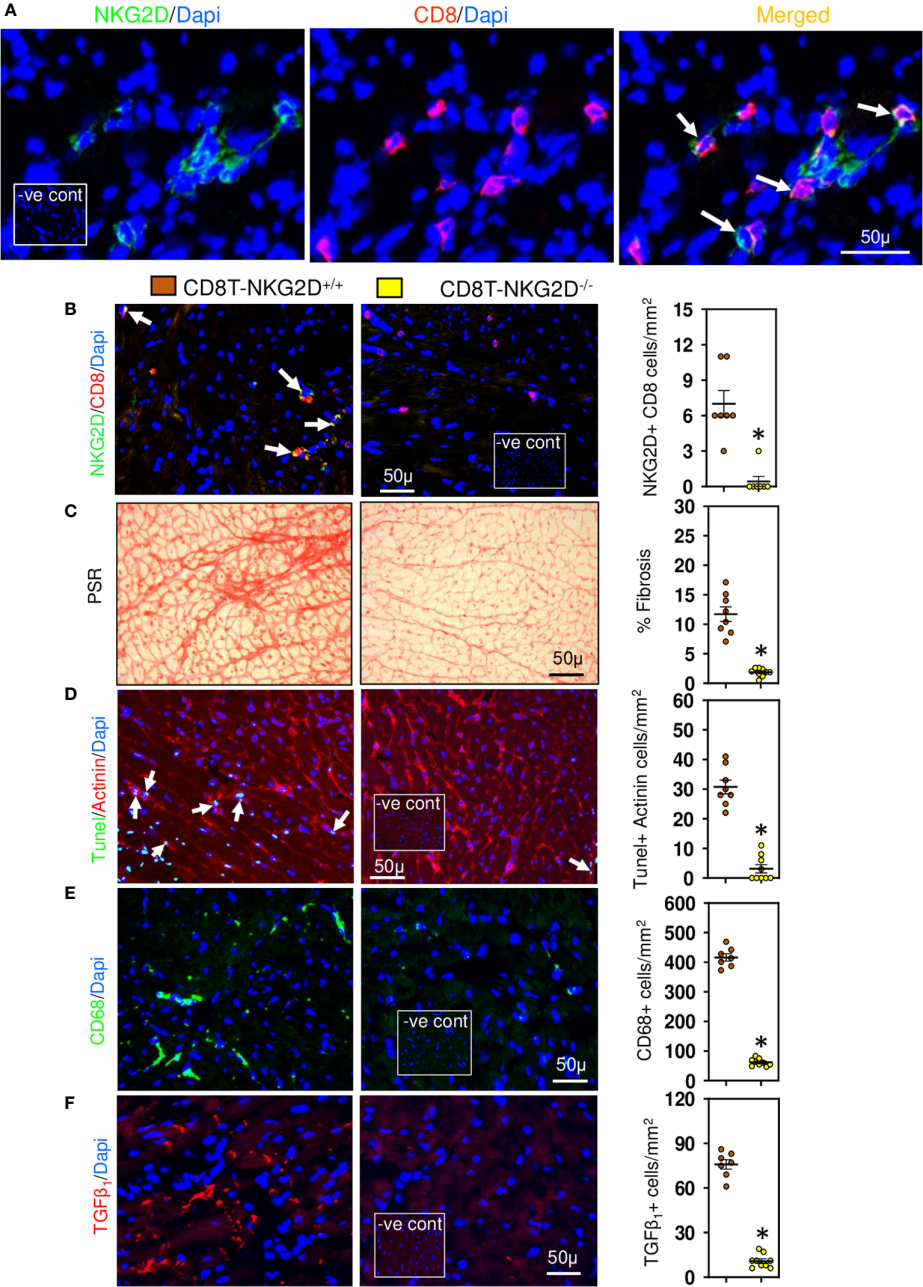
Cardiac CD8+ T cells require NKG2D receptors to promote fibrosis. **(A)** Photomicrographs indicating that CD8+ cells accumulated in LV of TAC mice express NKG2D receptor. **(B)** CD8+ T cells of TAC mice that underwent bone marrow transplantation with mixed bone marrow deficient in NKG2D (CD8T-NKG2D^-/-^) do not express NKG2D compared to controls (CD8T-NKG2D^+/+^). **(C)** LV fibrosis is attenuated in TAC mice with NKG2D-deficient CD8+ T cells. **(D)** TUNEL+ Actinin+ cardiomyocytes are greatly reduced in mice with NKG2D-deficient CD8+ T cells. **(E, F)** LV CD68+ macrophages and TGF-β1 expressing cells in TAC mice with NKG2D-deficient CD8+ T cells are markedly reduced. Results are means ± SEM with small circles representing data from individual mice. n=7-9/group. PSR; picrosirius, -ve control; no primary antibody control, scale bar represents 50µm. *P< 0.05 using two tailed Student’s t-test comparing to CD8T-NKG2D^+/+^.

Given the relationship between CD8+ T cells and cardiac apoptotic cells and the earlier proposal suggesting that CD8+ T cells promote fibrosis *via* innate mechanisms (17), we examined whether cardiac CD8+ T cells were cytotoxic and expressed innate NKG2D receptors; NKG2D receptors recognize stress-induced surface ligands and are sufficient to initiate a killing response (38). The majority of the CD8+ T cells in ventricular fibrotic areas expressed perforin (**Figure 3A**) and NKG2D (**Figure 4A**) in TAC mice. To determine if perforin was required for fibrosis, we generated mixed chimeric (CD8T-Pfp^-/-^) mice in which CD8+ T cells were made deficient in perforin (24, 39), transferring mixed bone marrow [80% from CD8β-deficient and 20% from perforin-deficient mice] into irradiated C57BL/6 mice; control (CD8T-Pfp^+/+^) mice received mixed bone marrow from CD8β-deficient (80%) and 20% from wild type mice (24, 39). In these mice CD8+ T cells are derived from bone marrow of either the perforin-deficient or wild type mice. Four weeks after bone marrow transplantation major lymphocyte populations in the spleen were comparable to those in non-irradiated C57BL/6 mice (**Figure 3B**). FACS analysis confirmed that in CD8T-Pfp^-/-^ mice, TCRβ+ CD8+ T cells did not express perforin (**Figure 3C**). Mixed chimeric mice were subjected to TAC surgery and effects on cardiac fibrosis examined 4-weeks later. Cardiac fibrosis was nearly abolished in mice with perforin-deficient CD8+ T cells (**Figure 3D**). Also cardiomyocyte apoptosis was markedly attenuated (**Figure 3E**). We assessed effects on ventricular macrophages and TGF-β1. Both macrophages and TGF-β1 were greatly reduced when perforin is deleted from CD8+ T cells (**Figures 3F, G**). In accordance with these findings, previous studies have reported that macrophages are the major apoptotic cell-clearing phagocytes in tissue homeostasis (40, 41) and their production of TGF-β1 is increased following phagocytosis of apoptotic cells (21, 42). To assess the importance of NKG2D we also generated mixed chimeric mice using bone marrow from CD8β-deficient, NKG2D-deficient and wild type mice and subjected the mice to TAC surgery. We confirmed that cardiac CD8+ T cells in hearts of chimeric mice did not express NKG2D (**Figure 4B**), however, NK/NKT and TCRγδ+ T cells accumulated in myocardial fibrotic areas expressed NKG2D receptor in TAC mice with NKG2D deficiency limited to CD8 T cells (**Figure S2**). Cardiac fibrosis in these mice was nearly abolished (**Figure 4C**); apoptotic cardiomyocytes were greatly reduced (**Figure 4D**) as were macrophage numbers and TGF-β1 expressing cells (**Figures 4E, F**). These findings indicate that CD8+ T cells are activated locally within the left ventricle by mechanisms dependent on the NKG2D receptor and use perforin to promote fibrosis.

### Cardiomyocyte RAE-1 and MCP-1 are increased *via* the cGAS-STING cytosolic DNA sensing pathway triggering CD8+ T cell cytotoxicity and interstitial fibrosis

**Figure 5:**
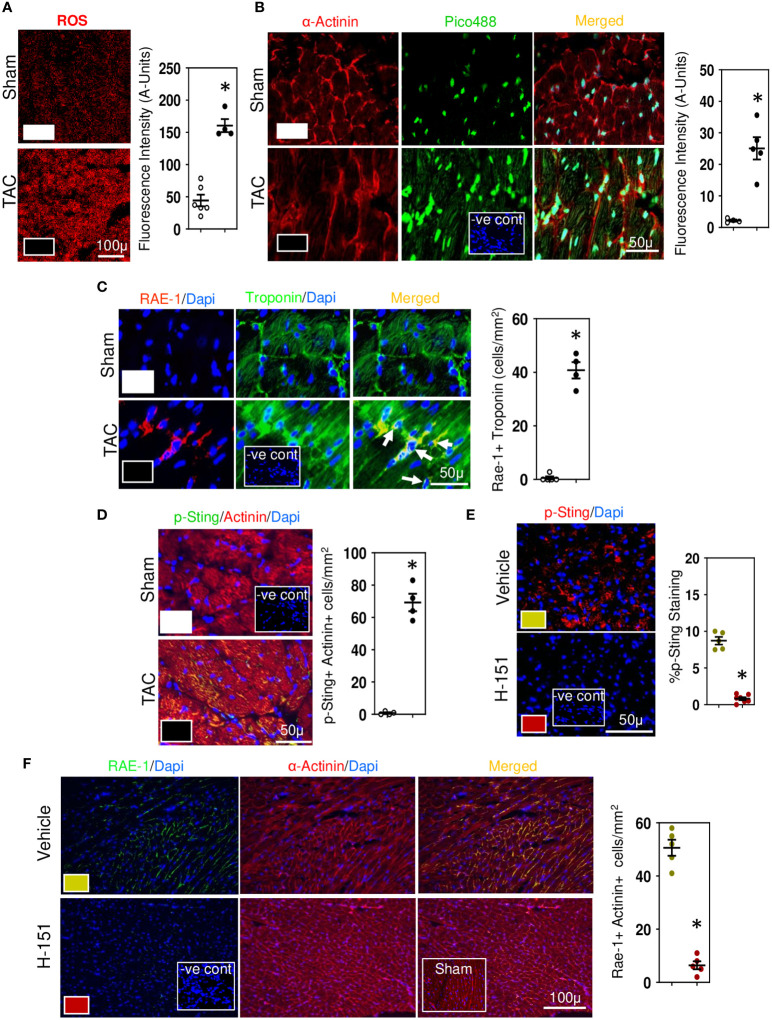
Cytosolic DNA and STING in stressed cardiomyocytes initiates the fibrotic signaling cascade. **(A)** Representative photomicrographs demonstrating increases in ROS in LV of TAC mice. **(B)** Photomicrographs demonstrating increases in cytosolic DNA in cardiomyocytes of TAC mice detected with Pico488. Representative photomicrographs demonstrating increases in RAE-1 expression in cardiomyocytes **(C)** together with increased phosphoSTING **(D)** during TAC-induced hypertension. Treatment with H-151, anti-STING small molecular inhibitor, reducesphosphoSTING expression **(E)** and prevents cardiomyocyte expression of RAE-1 expression **(F)** in LV of TAC mice. Results are means ± SEM with small circles representing data from individual mice. n=7-9/group. -ve control; no primary antibody control, sham; sham-operated, scale bar represents either 100µm or 50µm. *P<0.05 using two tailed Student’s t-test test comparing to either TAC or H-151.

**Figure 6:**
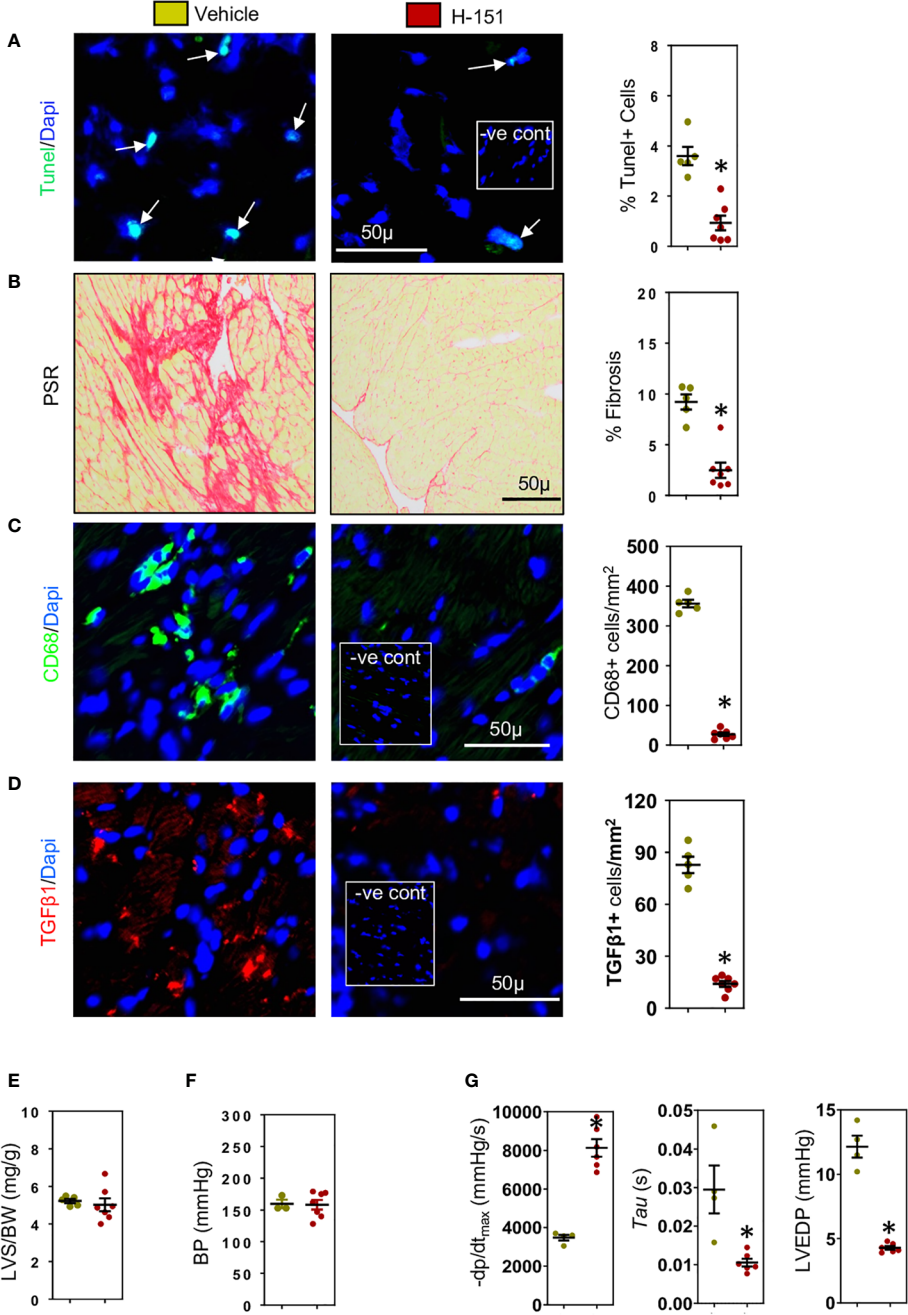
H-151, small molecule STING inhibitor, reduces cardiac fibrosis and improves left ventricular (LV) diastolic function. H-151 treatment markedly reduces **(A)** LV cell apoptosis and **(B)** LV fibrosis (PSR staining) in TAC mice. **(C)** LV CD68+ macrophages and **(D)** cells expressing TGF-β1 are greatly reduced in H-151 treated mice. **(E)** LV hypertrophy (LVS/BW) and **(F)** systolic blood pressure (SBP) are not affected by H-151 treatment. **(G)** LV diastolic function is improved by H-151 treatment. Results are means ± SEM with small circles representing data from individual mice. n=7-9/group. -ve control; no primary antibody control, scale bar represents 50µm *P< 0.05 using two tailed Student’s t-test, comparing to H-151.

We have previously shown that during development of cardiac fibrosis reactive oxygen species (ROS) are greatly increased (12). Increases in ROS promote mitochondrial DNA damage and its leakage into the cytoplasm (43) thus activating the cytoplasmic STING-TBK1-IRF3-IFN-β signaling pathway (44). As RAE-1 ligands which activate the NKG2D receptor are regulated *via* IRF3 (45), we investigated whether cardiac RAE-1 and the cGAS-STING pathway might be important for CD8+ T cell mediated interstitial fibrosis in the hypertensive heart. We confirm that high ROS levels in hypertensive hearts (**Figure 5A**) and using fluorescent DNA binding molecule pico488 (PicoGreen) (46), demonstrate the presence of cytosolic DNA in cardiomyocytes of hypertensive hearts (**Figure 5B**). Cardiomyocyte RAE-1 was also greatly elevated (**Figure 5C**); similarly, phosphoSTING was detectable in stressed cardiomyocytes (**Figure 5D**), indicating cGAS-STING DNA sensor pathway activation (43, 44). To determine whether this pathway was responsible for the RAE-1 expression, CD8+ T cell mediated apoptosis and fibrosis we treated TAC mice with H-151, a small molecule inhibitor that blocks STING plamitoylation, which is required for STING signaling (28, 47). Treatment inhibited cardiac STING signaling, indicated by the absence of phosphoSTING (**Figure 5E**); treatment also prevented RAE-1 expression (**Figure 5F**) and cardiac cell apoptosis (**Figure 6A**), resulting in reduced cardiac fibrosis (**Figure 6B**). Macrophages and TGF-β1 expressing cells were also greatly reduced (**Figures 6C, D**). Neither left ventricular hypertrophy nor blood pressure were affected (**Figures 6E, F**); left ventricular micromanometry indicated markedly improved left ventricular relaxation and diastolic function in H-151 treated mice (**Figure 6G**).

### H-151, small molecular anti-STING inhibitor, attenuates TBK-IRF3-NFkB signaling pathway

**Figure 7:**
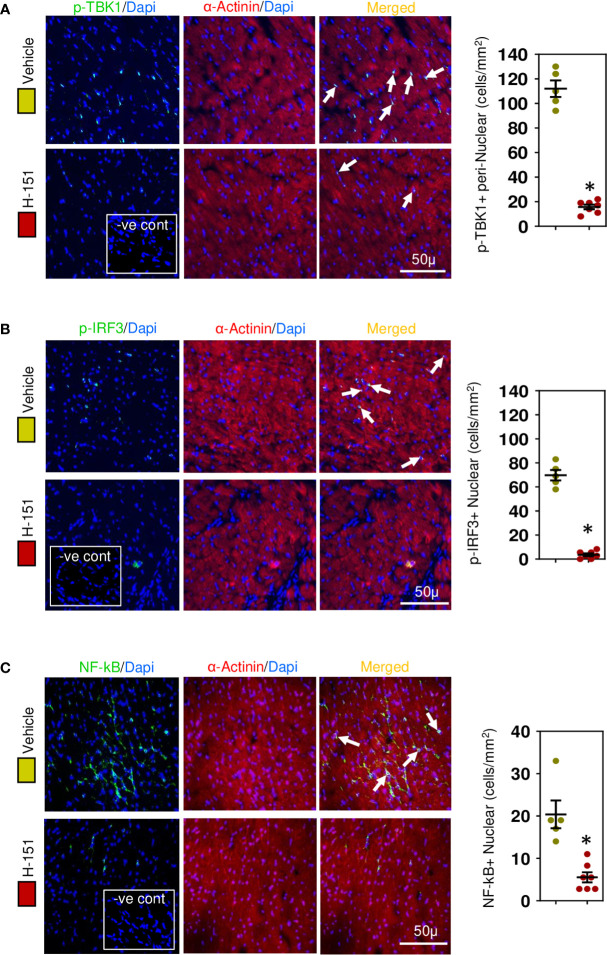
STING-IRF3-NFĸB signaling cascade is activated in cardiomyocytes during hypertensive fibrosis in TAC mice. **(A)** PhosphoTBK is increased in cardiomyocytes during fibrosis and prevented by H-151. **(B)** Nuclear phosphoIRF3 is increased in cardiomyocytes during fibrosis and inhibited by H-151. **(C)** Nuclear accumulation of NF-ĸB(p65) is increased during fibrosis and inhibited by H-151. Results are means ± SEM with small circles represent data from individual mice. n=5-7/group. -ve control; no primary antibody control, scale bar represents 50µm. *P< 0.05 using two tailed Student’s t-test, comparing to H-151.

**Figure 8:**
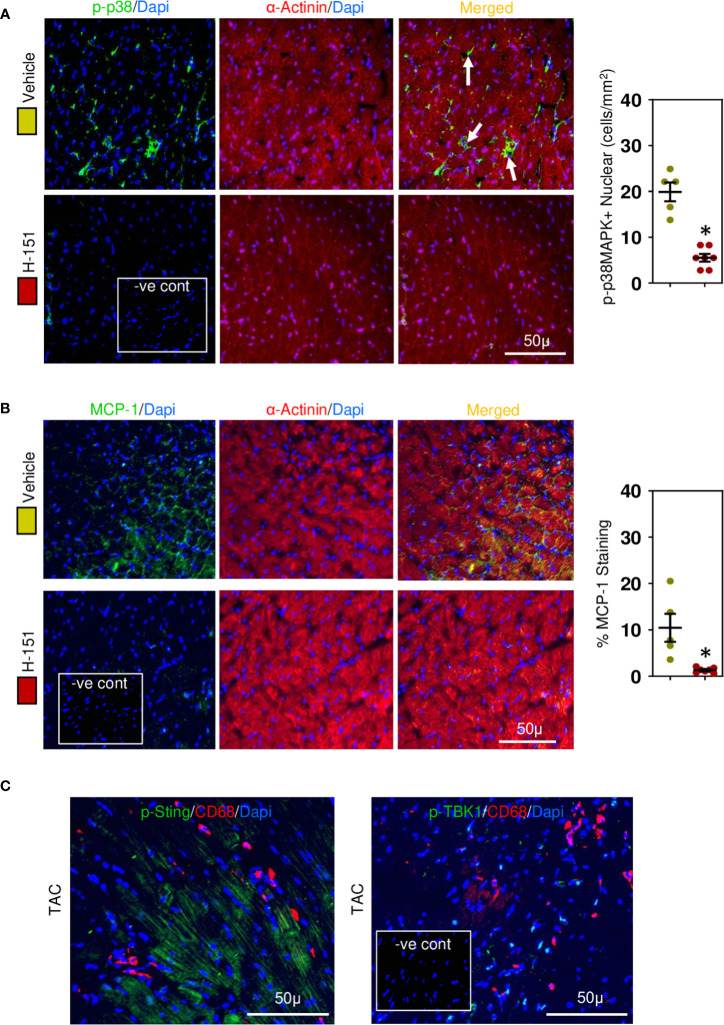
Cardiac phospho-p38MAPK and MCP-1 are upregulated in hypertensive cardiac fibrosis. **(A)** Nuclear phospho-p38MAPK is increased in cardiomyocytes during fibrosis development and inhibited by H-151. **(B)** Cardiomyocyte MCP-1 expression is increased during fibrosis development and prevented by H-151. **(C)** Macrophages associated with cardiac fibrosis do not express phosphoSTING or phosphoTBK1. Results are means ± SEM with small circles represent data from individual mice. n=5-7/group. -ve control; no primary antibody control, scale bar represents 50µm. *P< 0.05 from control TAC mice treated with vehicle only using two tailed Student’s t-test, comparing to H-151.

To confirm involvement of the STING, TBK-IRF3-NFkB signaling cascade we assessed the phosphorylation status of these molecules in relation to their intracellular location within cardiomyocytes. Phospho-TBK was apparent, localized in the perinuclear region (**Figure 7A**), whilst phospho-IRF3 appeared mostly nuclear (**Figure 7B**), as was NF-kB (**Figure 7C**) and phospho-p38 MAPK (**Figure 8A**). Because NF-kB and p38MAPK have been associated with increased MCP-1 (48, 49), a chemokine that not only attracts macrophages but also enhances their phagocytic activity (50), we examined its expression in cardiomyocytes. MCP-1 was increased during development of fibrosis and completely prevented by treatment with H-151 (**Figure 8B**) as were all other phosphorylation signals in the TBK-IRF3-NF-kB signaling cascade (**Figures 7**, **8A**); STING signaling was not apparent in macrophages associated with fibrosis (**Figure 8C**).

## Discussion

We show for the first time that two signals, one from overly stressed cardiomyocytes and the other from cytotoxic CD8+ T cells are required for development of hypertensive cardiac fibrosis; this interaction between the two cell types is critical for fibrosis. CD8+ T cells in the hypertensive heart appear to have a memory phenotype and are activated *via* interactions between NKG2D receptors expressed by the CD8+ T cells and RAE-1, the NKG2D ligand expressed by stressed cardiomyocytes. Bystander activation of CD8+ T cells is very common during viral infections (51) but here we show that it is also essential for initiation and development of hypertensive cardiac fibrosis. Fibrosis alters the cardiac electrical conduction system resulting in many deadly arrhythmias; it also increases ventricular stiffness which impairs cardiac function, thereby contributing to heart failure (6, 7).

Given that cardiomyocyte apoptosis is a frequent occurrence during hypertension in humans and mice (52–54), we hypothesized that overly stressed cardiomyocytes in hypertrophied hearts undergo apoptosis and as lost cardiomyocytes cannot be replaced, this will result in fibrosis. Indeed, we found that overly stressed cardiomyocytes initiate a cGAS-STING mediated signaling cascade resulting in RAE-1 expression which activates cytotoxic CD8+ T cells, thus initiating cardiomyocyte apoptosis which in turn activates macrophages and increases their expression of TGF-β1 (21). This notion explains why the genetic depletion of CD8+ T cells reduces cardiac fibrosis and heart failure in mice with pressure overload despite an initial macrophage accumulation (55). CD8+ T cells seem to recruit monocyte/macrophages and modulate them into cardioprotective phenotypes to maintain homeostasis before the commencement of myocardial stress (55). The interaction of TGF-β1 with cardiac fibroblasts stimulates collagen production resulting in fibrosis in hypertensive hearts (56). Whilst the amount of TGF-β produced by macrophages appears relatively small, TGF-β1 positively regulates its own expression and by interacting with fibroblasts greatly augments the TGF-β1 fibrotic signal (22, 23). Our study identifies the RAE-1/NKG2D interaction as a novel therapeutic target to prevent the gradual loss of cardiomyocytes in hypertensive hearts due to apoptosis and the cardiac fibrosis associated with HFpEF in hypertensive patients.

The mechanisms that initiate cardiac fibrosis in non-ischemic hearts are poorly defined. It has been suggested that dying cardiomyocytes release DAMPs which interact with pattern recognition receptors, in particular Toll-like receptors (TLRs) expressed by neighboring cardiomyocytes, fibroblasts, immune cells and parenchymal cells to initiate fibrosis (43); fibroblasts, macrophages and CD4+ T cells all contribute to cardiac fibrosis that develops in non-ischemic hypertensive hearts (16, 17, 56). Whilst multiple DAMPs are released by dying cardiomyocytes, particularly after myocardial infarction and include mitochondrial DNA (mtDNA), the chromatin protein high mobility group box 1 (HMGB1) and S100 proteins, their precise roles in fibrosis are still unclear (57). Exosomes derived from doxorubicin damaged cardiomyocytes can augment fibrosis but their relevance to non-ischemic hearts is also unclear (58). We demonstrate that an increase in cytosolic DNA in overly stressed cardiomyocytes is a key early event in fibrosis of non-ischaemic hypertensive hearts; this was confirmed by our demonstration of activation of the cGAS-STING cytosolic DNA sensing pathway. Activating this pathway in cardiomyocytes greatly increases expression of RAE-1, an NKG2D receptor ligand that is absent in non-stressed healthy cells and only induced upon cellular stress (59). RAE-1 expression was highly dependent on STING activation; RAE-1, an IRF3 responsive gene has previously been shown to be regulated by the STING-dependent DNA sensor pathway in lymphomas (45). We did not identify the source of the DNA but given the high levels of oxidative stress associated with the hypertensive hearts, it most likely represents mtDNA. We were not able to test this directly *in vivo* as cyclosporin A, which inhibits mitochondrial permeability transition (MPT) pore opening and mtDNA release (60), also inhibits CD8+ T cell activation (61). High ROS levels trigger MPT pore opening, resulting in release of mtDNA into the cytosol (60). Recently Hu et al. have also shown that deletion of the cytosolic DNA sensor cGAS contributes to cardiac pathology in mice with pressure overload-induced hypertension (62) but downstream signaling events responsible for cardiac pathology were not investigated; cGAS can have both STING-dependent and independent effects (63). Accumulation of OVA-specific CD8+ T cells in pressure overload-induced myocardium did not affect cardiac fibrosis (17, 18), suggesting the importance of their TCR-independent contribution in cardiac fibrosis. Our studies indicate that cytosolic DNA, acting as an intracellular DAMP initiates an inflammatory cascade by stimulating expression of RAE-1 there by activating NKG2D+CD8+ T cells and subsequent cellular/molecular signaling cascade resulting in fibrosis.

Our findings that depletion of CD8+ T cells reduced cardiac fibrosis independently of the etiology of the hypertension stimulated us to investigate in detail how these cells contributed to fibrosis. Previous studies utilizing CD8α chain genetic deletion have led to conflicting results indicating either a major role or no role in cardiac fibrosis associated with hypertensive hearts (16, 17). We focused on targeting CD8β chain with specific antibodies and also *via* genetic deletion and have previously used such a strategy to define the role of CD8+ T cells in atherosclerosis (32). We provide four independent lines of evidence that unequivocally demonstrate that CD8+ T cells are critical for cardiac fibrosis. Depleting CD8+ T cells using anti-CD8β antibodies greatly attenuated cardiac fibrosis in three different hypertensive mouse models indicating that it affects fibrosis independently of hypertension etiology. Furthermore, preventing help from CD4+ T cells markedly reduces CD8+ T cell numbers and fibrosis, effects consistent with the requirement of CD4+ T cells for many aspects of CD8+ T cell function, including their survival (37, 38) and generation of a cytotoxic phenotype (64). CD8+ T cells numbers in fibrotic regions were highly dependent on the presence of CD4+ T cells and the majority of CD8+ T cells expressed perforin. Perforin is essential for cell-mediated cytotoxicity enabling granzymes to enter target cells and induce apoptosis. Perforin deletion in CD8+ T cells greatly reduced cardiomyocyte apoptosis, TGF-β1 expression and cardiac fibrosis. Deletion of NKG2D in CD8+ T cells, which is critical for RAE-1 mediated CD8+ T cell activation had similar effects. Together these findings demonstrate a major role for cytotoxic CD8+ T cells in cardiac fibrosis (Graphical Abstract).

Treatments for HFpEF have limited efficacy and currently no drugs have been approved for the treatment of cardiac fibrosis. Experimentally, all currently proposed therapeutic strategies are focused on downstream rather than initiating events (65) and include cytokines, particularly TGF-β1, as well as targeting different cell types including fibroblasts and macrophages. TGF-β inhibitors are effective but toxic (65); similarly, the use of engineered T cells to destroy collagen producing fibroblasts although effective is also limited by toxicity (56) and therapeutically relevant approaches to target pro-fibrotic macrophages have not been developed. In this study we found that specifically targeting overly stressed cardiomyocytes that express RAE-1 is highly effective in preventing development of cardiac fibrosis in hypertensive hearts. Treatment with the STING inhibitor H-151 not only greatly reduced fibrosis but also reduces cardiomyocyte loss due to apoptosis by preventing RAE-1 expression and targeted killing by CD8+ T cells; this greatly reduces TGF-β1 expression; therapeutically it should be possible to target RAE-1/NKG2D interactions. Increases in myocardial stiffness together with reductions in relaxation are major contributors to diastolic dysfunction in HFpEF and can be directly assessed by measuring cardiac pressures (66). Inhibiting STING as well as CD8+ T cell deletion significantly improved diastolic function in hypertensive hearts, independently of blood pressure and left ventricular hypertrophy. Previously STING deletion using siRNA was associated with a reduction in left ventricular hypertrophy (67), contrasting with our finding that following inhibition of STING palmitoylation with H-151 left ventricular hypertrophy is unaffected. Unfortunately, siRNA details were not provided. Chemical modification such a 2”-O-methyl modification of a single position in the siRNA guide strand is required to reduce most siRNA off-targeting silencing (68). STING inhibition using H-151 did not affect blood pressure, consistent with our finding of no effect on left ventricular hypertrophy. Together our findings indicate that STING signaling in overly stressed cardiomyocytes is critically important for development of hypertensive fibrosis. Whether STING inhibition is a suitable therapeutic strategy to prevent cardiac fibrosis remains to be determined, as inhibition can compromise immune responses to viral infections as well as cancer (69).

In summary, our data demonstrate that cytoplasmic DNA released in overly stressed cardiomyocytes in hypertensive hearts induces expression of the NKG2D ligand RAE-1 *via* the cGAS-STING pathway, which activates NKG2D+perforin+CD8+ T cells. These CD8+ T cells selectively target RAE-1 expressing cardiomyocytes inducing apoptosis. STING signaling in stressed cardiomyocytes also increases MCP-1 expression attracting macrophages and promoting phagocytosis which initiates TGF-β1 expression and development of interstitial cardiac fibrosis. Therapeutically, targeting RAE-1 to prevent RAE-1-NKG2D interactions may be a useful strategy to prevent interstitial fibrosis causing diastolic dysfunction in HFpEF.

## Data availability statement

The raw data supporting the conclusions of this article will be made available by the authors, without undue reservation.

## Ethics statement

The animal study was reviewed and approved by Animal Ethics Committee at the Alfred Research Alliance, Melbourne, Australia.

## Author contributions

TK, B-HT, KP and AB conceived and designed experiments. TK, PK, KB and AC performed experiments and analyzed data. TK drafted and TK, KB, B-HT, KP and AB wrote the manuscript. All authors contributed to the article and approved the submitted version.

## Funding

This work was supported by grants (APP1106151 to AB, B-HT, TK, APP1162251 to AB and B-HT) from the National Health and Medical Research Council of Australia.

## Conflict of interest

The authors declare that the research was conducted in the absence of any commercial or financial relationships that could be construed as a potential conflict of interest.

## Publisher’s note

All claims expressed in this article are solely those of the authors and do not necessarily represent those of their affiliated organizations, or those of the publisher, the editors and the reviewers. Any product that may be evaluated in this article, or claim that may be made by its manufacturer, is not guaranteed or endorsed by the publisher.

## Supplementary material

Supplementary Figure 1 CD8+ T cells promote cardiac fibrosis in mice with 2K1C-renal hypertension and with DOCA-salt hypertension. **(A)** Data showing depletion of CD8αβ+ T cells in LV by anti-CD8β depleting antibodies compared with control mice with 2K1C-renal hypertension. **(B)** Systolic blood pressure (SBP) is unaffected by CD8β antibody treatment. **(C)** LV hypertrophy (LVS/BW) in 2K1C mice is unaffected by antibody treatment. **(D)** Photomicrographs showing the reduction in LV fibrosis in 2K1C mice associated with CD8αβ+ T cell depletion. **(E)** CD8αβ+ T cell depletion improves LV diastolic function in 2K1C-hypertensive mice. **(F)** CD8αβ+ T cell depletion reduces cardiac cell TUNEL+ apoptotic cells. **(G)** CD8β antibody treatments reduces cardiac CD8+ T cells in mice with DOCA-salt hypertension compared to controls. **(H)** Systolic blood pressure and **(I)** LV hypertrophy are unaffected by CD8+ T cell depletion in mice with DOCA-salt hypertension. **(J)** Photomicrographs showing greatly reduced cardiac fibrosis in DOCA-salt hypertensive mice treated with anti-CD8β depleting antibodies. **(K)** LV diastolic function is improved following CD8+ T cell depletion in DOCA-salt hypertensive mice. **(L)** LV apoptotic cells are reduced following CD8+ T cell depletion in DOCA-salt mice. n=4-11/group. Results are means ± SEM with small circles representing data from individual mice. scale bar represents 50µm. *P < 0.05 using two-tailed Student’s t-test, comparing to either 2K1C+IgG or DOCA+IgG.

Supplementary Figure 2 NK/NKT and TCRγδ T cells express NKG2D receptors in TAC CD8T-NKG2D^+/+^ mice. Mice were subjected to mixed chimeric approach (see text for detail) to generate mice with CD8+ T cell-specific NKG2D deficiency (CD8T-NKG2D^-/-)^. Representative photomicrographs demonstrating the expression of NKG2D in NK1.1+ and TCRγδ+ cells accumulated in fibrotic areas of left ventricles in TAC CD8T-NKG2D^-/-^ mice as well as TAC CD8T-NKG2D^+/+^ control mice. scale bar represents 50µm.
