# Supplementary figures and images for: Crosstalk between cytotoxic CD8+ T cells and stressed cardiomyocytes triggers development of interstitial cardiac fibrosis in hypertensive mouse hearts
Source: Front Immunol. 2022 Nov 22;13:1040233. doi: 10.3389/fimmu.2022.1040233 (PMC9724649; doi:10.3389/fimmu.2022.1040233)

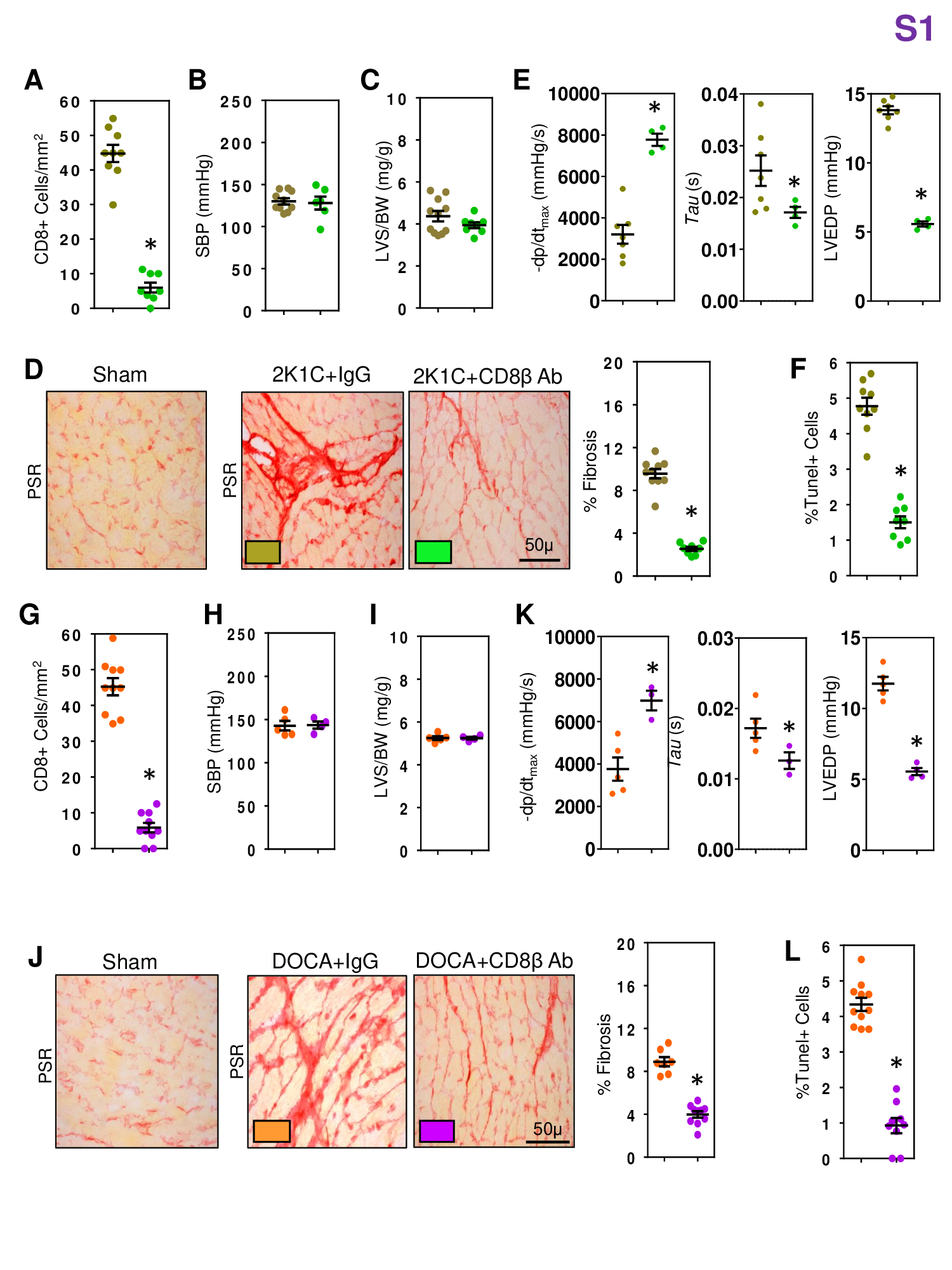

Supplement: Supplementary Figure 1 — CD8+ T cells promote cardiac fibrosis in mice with 2K1C-renal hypertension and with DOCA-salt hypertension. (A) Data showing depletion of CD8αβ+ T cells in LV by anti-CD8β depleting antibodies compared with control mice with 2K1C-renal hypertension. (B) Systolic blood pressure (SBP) is unaffected by CD8β antibody treatment. (C) LV hypertrophy (LVS/BW) in 2K1C mice is unaffected by antibody treatment. (D) Photomicrographs showing the reduction in LV fibrosis in 2K1C mice associated with CD8αβ+ T cell depletion. (E) CD8αβ+ T cell depletion improves LV diastolic function in 2K1C-hypertensive mice. (F) CD8αβ+ T cell depletion reduces cardiac cell TUNEL+ apoptotic cells. (G) CD8β antibody treatments reduces cardiac CD8+ T cells in mice with DOCA-salt hypertension compared to controls. (H) Systolic blood pressure and (I) LV hypertrophy are unaffected by CD8+ T cell depletion in mice with DOCA-salt hypertension. (J) Photomicrographs showing greatly reduced cardiac fibrosis in DOCA-salt hypertensive mice treated with anti-CD8β depleting antibodies. (K) LV diastolic function is improved following CD8+ T cell depletion in DOCA-salt hypertensive mice. (L) LV apoptotic cells are reduced following CD8+ T cell depletion in DOCA-salt mice. n=4-11/group. Results are means ± SEM with small circles representing data from individual mice. scale bar represents 50µm. *P < 0.05 using two-tailed Student’s t-test, comparing to either 2K1C+IgG or DOCA+IgG. [file Image_1.tiff]

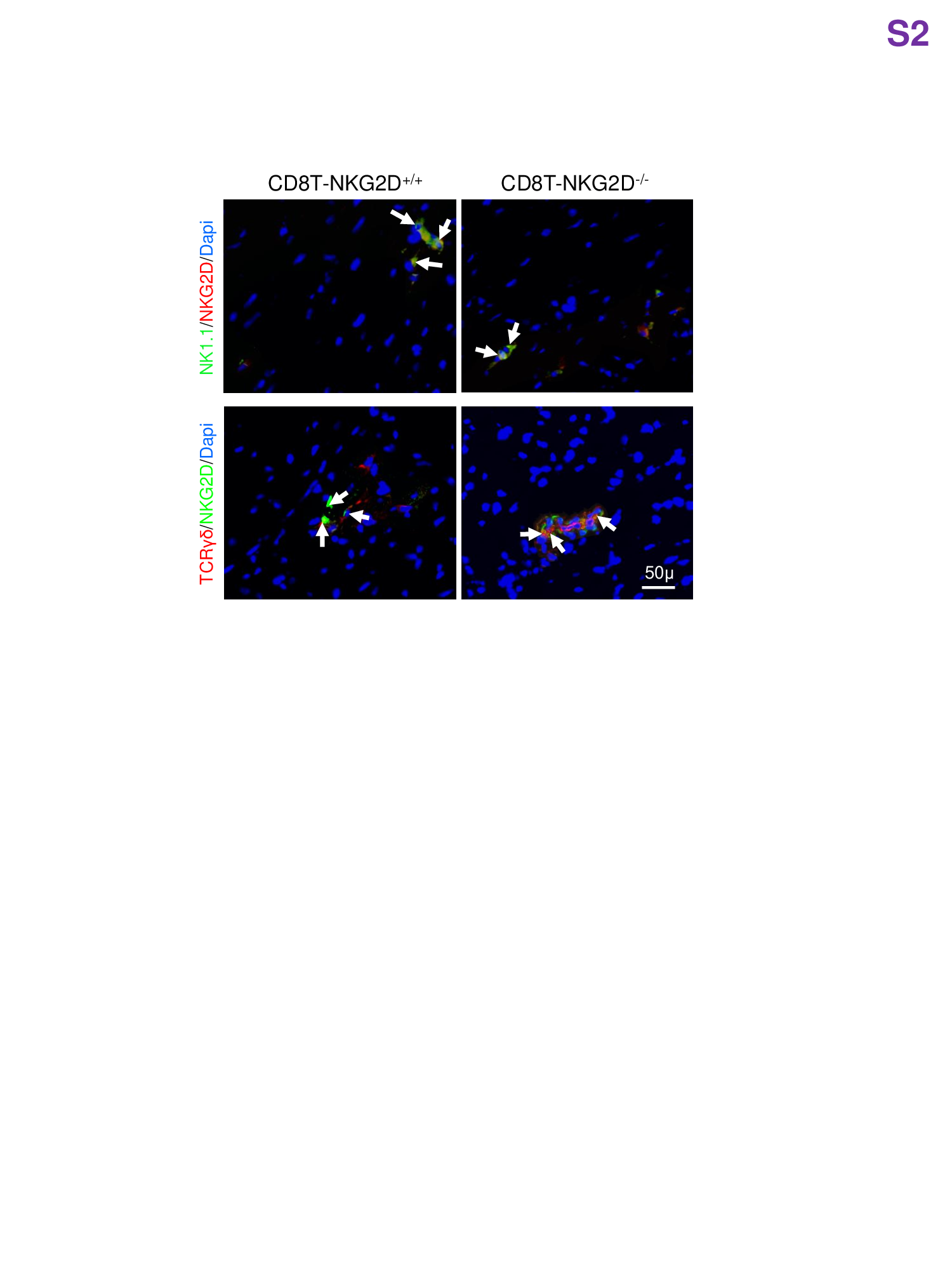

Supplement: Supplementary Figure 2 — NK/NKT and TCRγδ T cells express NKG2D receptors in TAC CD8T-NKG2D+/+ mice. Mice were subjected to mixed chimeric approach (see text for detail) to generate mice with CD8+ T cell-specific NKG2D deficiency (CD8T-NKG2D-/-). Representative photomicrographs demonstrating the expression of NKG2D in NK1.1+ and TCRγδ+ cells accumulated in fibrotic areas of left ventricles in TAC CD8T-NKG2D-/- mice as well as TAC CD8T-NKG2D+/+ control mice. scale bar represents 50µm. [file Image_2.tiff]
